# Gut Bacterial Communities of *Lymantria xylina* and Their Associations with Host Development and Diet

**DOI:** 10.3390/microorganisms9091860

**Published:** 2021-09-01

**Authors:** Qiuyu Ma, Yonghong Cui, Xu Chu, Guoqiang Li, Meijiao Yang, Rong Wang, Guanghong Liang, Songqing Wu, Mulualem Tigabu, Feiping Zhang, Xia Hu

**Affiliations:** 1 International Joint Laboratory of Forest Symbiology, College of Forestry, Fujian Agriculture and Forestry University, Fuzhou 350000, China; mqy952107954@126.com (Q.M.); cuiyonghong2021@126.com (Y.C.); chuxu1996@126.com (X.C.); guoqiangli2021@126.com (G.L.); meijiaoyang2021@126.com (M.Y.); wangrongbl0501@126.com (R.W.); fjlhg@126.com (G.L.); dabinyang@126.com (S.W.); fpzhang1@163.com (F.Z.); 2 Key Laboratory of Integrated, Pest Management in Ecological Forests, Fujian Agriculture and Forestry University, Fuzhou 350000, China; 3 Southern Swedish Forest Research Center, Faculty of Forest Science, Swedish University of Agricultural Sciences, SE-230 53 Alnarp, Sweden; Mulualem.Tigabu@slu.se

**Keywords:** gut microbiota, development, diet, *Lymantria xylina*

## Abstract

The gut microbiota of insects has a wide range of effects on host nutrition, physiology, and behavior. The structure of gut microbiota may also be shaped by their environment, causing them to adjust to their hosts; thus, the objective of this study was to examine variations in the morphological traits and gut microbiota of *Lymantria xylina* in response to natural and artificial diets using high-throughput sequencing. Regarding morphology, the head widths for larvae fed on a sterilized artificial diet were smaller than for larvae fed on a non-sterilized host-plant diet in the early instars. The gut microbiota diversity of *L. xylina* fed on different diets varied significantly, but did not change during different development periods. This seemed to indicate that vertical inheritance occurred in *L. xylina* mutualistic symbionts. *Acinetobacter* and *Enterococcus* were dominant in/on eggs. In the first instar larvae, *Acinetobacter* accounted for 33.52% of the sterilized artificial diet treatment, while *Enterococcus* (67.88%) was the predominant bacteria for the non-sterilized host-plant diet treatment. Gut microbe structures were adapted to both diets through vertical inheritance and self-regulation. This study clarified the impacts of microbial symbiosis on *L. xylina* and might provide new possibilities for improving the control of these bacteria.

## 1. Introduction

The symbiotic association between bacteria and insects is a well-known and universal phenomenon, and is important in the biological processes of host insects. In this symbiotic relationship, the gut microbiota is essential for maintaining insect health [1,2]. The intestinal bacterial community usually provides metabolic benefits to the host through the production of digestive enzymes and vitamins, thereby improving nutrient absorption [3]. In stinkbugs of the family Plataspidae, gut bacteria were vertically transmitted as a ‘symbiont capsule’. When the ‘symbiont capsule’ was removed, the host insect showed high mortality rates [4]. The intestinal symbiotic bacteria of the oriental fruit fly *Bactrocera dorsalis* (Hendel) adjust the ecology and physiology of the host insect and even help host insects improve their resistance to insecticides [5].

In recent microbial studies, diet and environment were considered to be the main factors in the formation of gut microbiota in insects [6]. For example, xylophagous insects had more complex gut microbiota communities, while insects that fed on sap, such as aphids and psyllids, had the least complex gut microbiota communities [7]. The gut microbiota communities of termites changed when fed on corn stover or wood diets [8]. Diverse diets could rapidly and greatly change an insect’s gut microbiota [1]. For instance, *Lymantria dispar asiatica* larvae showed great differences in their gut microbiota after the pH values of their food were changed [9]. *Lymantria xylina* Swinhoe larvae feeding on an artificial diet or host plants separately resulted in larger body size among the *L. xylina* larvae in the host-plant feeding group [10].

Lepidoptera is the most important order of agricultural pest insects in the world, and a highly diverse group of insects [11]. Casuarina moth (*L. xylina*) larvae are highly polyphagous, and their host range is gradually expanding because of their complex and rich microbial community [12]. In addition, *L. xylina* has been particularly harmful to *Casuarina equisetifolia* forests in the eastern coastal areas of China in recent decades [13]. Among the 157,424 recognized lepidopteran species, less than 0.1% have had their bacterial associates revealed. Therefore our knowledge of bacterial associates in Lepidoptera is still limited [14]. We hypothesized that *L. xylina* from all samples in this study would share specific bacteria, and that the bacterial community structure would have some associations with the organisms’ stage of development and diet; thus, the objective of this study was to examine the variations in morphological traits and gut microbiota of *L. xylina* in response to natural and artificial diets.

## 2. Materials and Methods

### 2.1. Insect Collection

All *L. xylina* egg masses were collected in the casuarina shelter forest in Matui Comprehensive Experimental Area from July 2017 to June 2018. The station was located on the west coast of the Taiwan Strait (25.53° N, 119.71° E) in Pingtan, Fujian, China. *L. xylina* egg masses were removed from infested *C. equisetifolia* and transported to the laboratory. Egg masses were kept at 9 °C until they hatched.

### 2.2. Effect of Symbiotic Bacteria on L. xylina Development

The potential effect of gut microbiota on *L. xylina* development was studied using aseptic feeding experiments. Collected eggs were assigned to feed in two diet-groups: a non-sterilized *C. equisetifolia* branch (NSC) and a sterilized artificial diet mainly composed of a *C. equisetifolia* branch (SAC). For the sterilized artificial feeding experiment (feeding on SAC), eggs were disinfected by an incubation in 75% ethanol for 3 min, then washed with sterile water 2–3 times to remove surface microbiota. To simulate natural feeding (feeding on NSC), other eggs were unsterilized. The eggs from the different treatment groups were transferred to high-temperature sterilized (121 °C, 20 min) closed transparent plastic (Polycarbonate) boxes (the upper mouth of the plastic boxes measured 17.2 × 11.3 cm, while the lower mouth measured 12.2 × 8.5 × 8.1 cm), and were stored in an incubator (MIR-154-PC Incubator, Panasonic, Kadoma, Japan) at 26 °C and 80% relative humidity with a photoperiod of 12:12 (light/dark) to break the egg diapause. After the hatching of first instar larvae, they were grouped into 5 boxes/diet groups, each containing 50 larvae from one of the two treatment groups. These were maintained in different incubators under the same conditions with different diets. In the NSC treatment, the first instar larvae were transferred to a breathable transparent plastic box, and the NSC diet was changed daily. In the SAC treatment, the first instar larvae were transferred to a new high-temperature sterilized transparent closed plastic box and fed on SAC. In order to maintain ventilation and sterility, air exchange and feed changeover were carried out every day in the sterile environment of the AlphaClean1300 system (AlphaClean1300, HealForce, Shanghai, China). All groups were fed until pupation. The artificial SAC diet was introduced in a sterile environment after high-temperature sterilization (121 °C, 20 min) of the food, while the diet formula was the modified feed A diet described by Shen and colleagues: sawdust from *C. equisetifolia* branches (147 g), sorbic acid (2 g), vitamin mix (vitamins A, E, B1, B2, B3, B5, B6, B9, B12, C, H, chloride, inositol) (10 g), P-nitro benzoic acid (1 g), ferric citrate (0.0428 g), agar (15 g), and distilled water (1 L) [10]. Molting was taken as the age classification standard. Pictures were taken of the first molting larvae by fluorescence stereomicroscope (M205FA, Leica, Wetzlar, Germany). The head widths and body lengths of *L. xylina* larvae were measured by ‘Measurement Tool’ (Leica Application Suite Version 4.8.0). A minimum of 10 larvae per sample were measured for biological repetitions.

### 2.3. DNA Extraction, Bacterial 16s rRNA Gene Amplification, and High-Throughput Sequencing

The insect bodies were disinfected with 70% ethanol for 3 min, and then washed twice in asepsis. In addition, the method proposed by Hu et al. was used to dissect and collect intestinal bacteria [15], whereby 10 μL 10 mM sterile phosphate-buffered saline (138 mM NaCl and 2.7 mM KCl, pH 7.4) was placed in a sterile culture dish. The posterior end of the larvae was cut off using alcohol-sterilized surgical scissors. The gut was pulled out under a stereomicroscope with sterile insect pins, transferred into a 1.5 mL centrifuge tube, and stored at −80 °C (MDF-DU502VXL refrigerator, Panasonic, JPN) for subsequent DNA extraction. All procedures were performed in a sterile environment. Non-sterilized eggs, disinfected eggs, and guts of larvae (seven instars), pupae (5th day), and adults were transferred into individual tubes for DNA extraction separately. There were 3 samples in each developmental stage, and a total of 57 samples were obtained (one gut = one sample). The bacterial DNA of all samples was extracted by the CTAB method. The DNA extraction of pupal gut bacteria fed on SAC failed. Purified bacterial DNA was diluted to 1 ng/μL with ddH_2_O. We then used the diluted genomic DNA as a template, and the V4 regions of the bacteria 16S ribosomal RNA genes were amplified by PCR using the following primers with barcodes: 515F 5′-GTGCCAGCMGCCGCGGTAA-3′ and 806R 5′-GGACTACHVGGGTWTCTAAT-3. PCR reactions were carried out in 30 µL reaction solution with 15 µL of Phusion High-Fidelity PCR Master Mix (New England Biolabs, Beijing, China), 0.2 µM of forward and reverse primers, and about 10 ng template DNA. Thermal cycling consisted of initial denaturation at 98 °C for 1 min, followed by 30 cycles of denaturation at 98 °C for 10 s, annealing at 50 °C for 30 s, elongation at 72 °C for 30 s, and finally at 72 °C for 5 min. PCR products were purified, and the quality was assessed using a Qubit 2.0 Fluorometer. The amplificates were sequenced on an Ion S5TM XL (Thermofisher, Shanghai, China) platform was used. Samples were examined for contamination with a blank PCR control (sterile water) before sequencing. The library was constructed and sequenced by Novogene Technology (Fujian, China).

### 2.4. Division of Larval Instars

Regression analysis was performed on the indices measured on each instar larva to verify the rationality of instar division. The Brooks and Crosby indices were calculated according to Dyar’s law and Crosby’s growth law [16]. The Brooks index was calculated using the following formula:

Brooks index = *X*_n_/*X*_n−1_, (1)

where n represents the larvae’s instar; *X*_n_ and *X*_n−1_ represent the means of the body length and head capsule width of the n^th^ instar and n−1^th^ instar larvae, respectively. The Crosby index was calculated using the following formula:

Crosby index = (*b*_n_ − *b*_n−1_)/*b*_n−1_, (2)

where n represents the larvae’s instar; *b*_n_ and *b*_n−1_ represent the Brooks index values of the n^th^ and n−1^th^ instar larvae, respectively [17]. When the coefficient of variation is <20% or the Crosby index is <10%, the instar division is reliable.

### 2.5. Bioinformatic Analysis

To obtain raw reads, low-quality fragments, the barcode, and primer sequences were cut and filtered using Cutadapt (V1.9.1) [18]. The raw reads sequences were compared with the species annotation database to detect and remove the chimera sequence in order to obtain clean reads [19,20]. All clean reads of all samples were clustered using Uparse [21]. Sequences were clustered into operational taxonomic units (OTUs) with 97% similarity using Mothur. Annotation of the sequences was performed in the SSUrRNA database (threshold of 0.8–1) to obtain taxonomic information [22]. OTU abundance information was normalized using a standard sequence number corresponding to the sample with the least sequences. All Illumina reads were deposited in the NCBI Short Read Archive database (accession number: PRJNA660917).

### 2.6. Gut Microbiota Composition of L. xylina on Different Diets

From the sequence results, we analyzed the complexity of the species diversity for each sample through 4 indices, namely the Chao1, Shannon, Simpson, and ACE indices. All of the indices used for our samples were calculated with QIIME (Version 1.7.0). The Mothur method and the SSUrRNA database [23] of SILVA132 (http://www.arb-silva.de/ (accessed on 25 March 2019)) [24] were used to perform species annotation analysis (with threshold values of 0.8~1) to obtain taxonomic information and classification level counts in order to determine the community composition of each sample. This was conducted to show the diversity of the gut microbiota at the phylum and genus levels in *L. xylina* fed on different diets, using GraphPad Prism software (Version 6.01). This study focused on the most richly represented taxa, whereby the top 10 phyla and genera were selected for bacterial community analysis based on their relative abundance. Sequences from other than the first 10 phyla or genera were referred to as ‘others’. We also compared beta diversity values to evaluate differences between samples in terms of species-diversity complexity using the weighted Unifrac distance metric and QIIME software (Version 1.7.0). We used LEfSe analysis to analyze the differences in species abundance data between groups using the rank sum test method, implemented a dimensionality reduction through LDA to evaluate the impacts of different species, and finally drew a histogram of the distribution of LDA values and evolutionary clades of different species. Principal coordinate analysis (PCoA) was performed to obtain principal coordinates from complex, multidimensional data.

### 2.7. Functional Annotation

We extracted the KEGG database prokaryotic whole genome 16S rRNA gene sequence and then applied the BLASTN algorithm to compare it to the SILVA SSU Ref NR database (BLAST bitscore > 1500) to establish the correlation matrix. The KEGG database prokaryotic annotation process was performed using UProC and PAUDA. The genomic function information was calibrated to the SILVA database to allow the SILVA database function annotation. Sequencing samples were clustered using the SILVA database sequence as the reference sequence, and then functional annotation information was obtained. According to the annotation results, we selected the grouping at the highest abundance of each annotation level to generate a functional relative abundance column stacking chart, so as to visually view the relatively high abundance of each sample at different annotation levels of functions and their proportions.

### 2.8. Statistical Analyses

The Shapiro–Wilk test was used to evaluate the normality of the data. The Kruskal–Wallis test was used for analysis of potentially significant differences for non-normality data, while the Student’s *t*-test was used for normality data. Multi-sample comparisons were performed in accordance with the normal distribution through one-way analysis of variance (ANOVA). These statistical tests were analyzed using SPSS (Version 21.0.0).

## 3. Results

### 3.1. Division of Larval Instars

A total of 214 *L. xylina* larvae were measured, in which process the head capsule widths and body length values were recorded. According to the variation index, the coefficient of variation was <20% for the head capsule width, but was >20% for the body length in the fifth and seventh instar (Table 1 and Table A1); therefore, head capsule width was more reliable as an indicator of the larval instar of L. xylina than body length. Except for the fifth instar, the body lengths were significantly different between larvae fed on NSC and and those fed on SAC (*p* < 0.05). The head capsule widths did not differ significantly (*p* > 0.05) between the NSC and SAC treatments for the first and second instar larvae, while from the third to fifth instars, the head capsule widths of the L. xylina larvae fed on NSC were significantly higher than the larvae fed on SAC (*p* < 0.05). There were no significant differences in the width of the head capsules of the sixth instar L. xylina larvae between NSC and SAC diets (*t*-test, *p* = 0.605) (Table 1).

### 3.2. Bacterial Community Structures and OTUs

**Figure 1:**
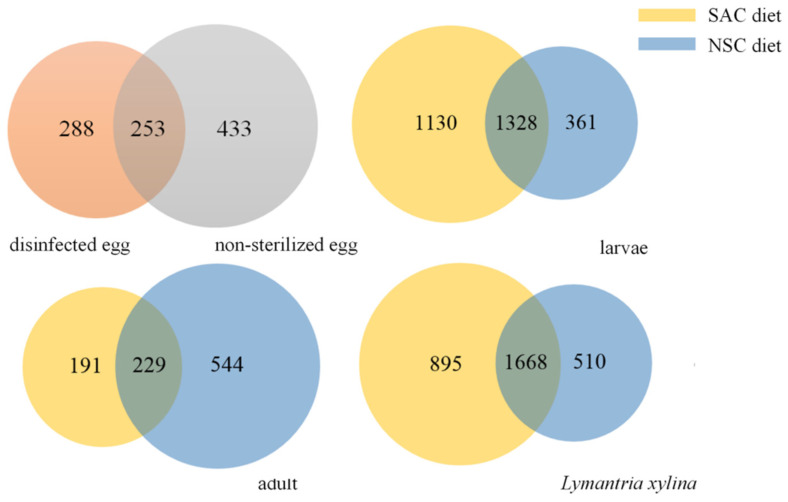
OTUs of egg and gut microbiota of *L. xylina* fed on SAC and NSC. SAC represents *L. xylina* fed on a sterilized artificial diet mainly composed of a *C. equisetifolia* branch, while NSC represents *L. xylina* fed on a diet of non-sterilized *C. equisetifolia* branch.

To describe the intestinal bacterial community structure of *L. xylina*, 57 samples were sequenced. After quality control, an average of 75,329 valid data points were obtained for each sample. The quality control efficiency reached 94.49%. This sequence was aggregated into OTUs with 97% identity, and a total of 3073 OTUs were obtained. Pairs of samples between the different diets at each stage shared some common OTUs, with *L. xylina* larvae sharing the most OTUs with 1328 OTUs (Figure 1). There was no significant difference between *L. xylina* eggs and adults’ OTU numbers on either diet (*p* > 0.05), while the OTUs of *L. xylina* larvae fed on NSC were lower than those that received the SAC treatment. The total number of OTUs for *L. xylina* fed on SAC (2563 OTUs) was significantly higher than on the NSC diet (2178 OTUs) (*t*-test, df = 52, *p* = 0.009), with 1668 OTUs in common.

**Figure 2:**
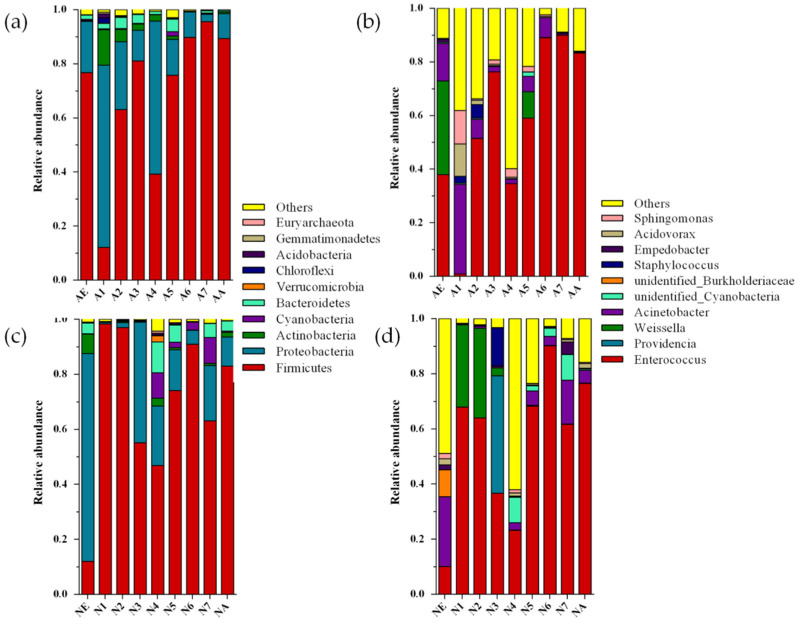
The relative abundance of bacteria in the gut microbiota from *L. xylina* fed different diets, at the phylum and genus levels: (**a**) at the phylum level for *L. xylina* fed on SAC; (**b**) at the phylum level for *L. xylina* fed on NSC; (**c**) at the genus level for *L. xylina* fed on SAC; (**d**) at the phylum level for *L. xylina* fed on NSC. Note: A1–A7: the first to seventh instars for larvae of *L. xylina* under the SAC treatment; N1–N7: the first to seventh instars for larvae of *L. xylina* under the NSC treatment.

The 3073 OTUs reported in this study were assigned to phylum 38, class 58, order 131, family 246, genus 619, and species 432. At the phylum level, Firmicutes was predominant, with a mean relative frequency of 66.4%, followed by Proteobacteria (25.9%) and Actinobacteria (20.6%). At the genus level, the *Enterococcus* genus was predominant, with a mean relative frequency of 55.0%, followed by *Acinetobacter* (11.0%) and *Weissella* (6.6%). The proportions of *Enterococcus* in the seven instars of larvae fed on NSC were 67.9%, 63.9%, 36.7%, 23.2%, 68.2%, 90.2%, and 61.6%, while for larvae fed on NSC, the values were 0.8%, 51.4%, 76.3%, 34.6%, 59.0%, 88.98%, and 89.90%. Both groups showed a trend of decreasing first and then increasing; however, for larvae fed on SAC, the most abundant gut bacteria of the first instar larvae were *Acinetobacter* (33.5%), followed by *Sphingomonas* (12.5%) and *Acidovorax* (12.1%) (Figure 2).

### 3.3. The Diversity Differences in Bacterial Communities during the Different Development Stages of L. xylina

According to the alpha diversity results, across development stages, the gut microbiota diversity for *L. xylina* first increased and then decreased. The *L. xylina* adults had the smallest bacterial populations (Chao1 = 333.503) of the different development stages. In the larval stage, the fourth instar larvae had the most abundant bacterial communities and the largest bacterial population (Chao1 = 531.653, Shannon = 4.484), while the sixth instar larvae had the least-abundant bacterial communities (Chao1 = 279.847) (Table A2). The relative abundance levels of OTU members were different during the different *L. xylina* development stages on both SAC and NSC diets. The proportion of Proteobacteria (47.23%) was larger than Firmicutes (44.33%) for *L. xylina* eggs; however, during the development of the larvae into adults, the proportion of Firmicutes gradually increased to become the most dominant population and reached the highest value at the sixth instar (90.29%), which was significantly higher than in the adults and in larvae at other instar points (*t*-test, df = 52, *p* = 0.000) (Table A3). At the genus level, the dominant bacteria from eggs were *Enterococcus* (23.98%), followed by *Acinetobacter* (19.61%) and *Weissella* (17.48%), while the proportion of *Enterococcus* increased (except for in the fourth instar larvae) with the progression of instars for the *L. xylina* larvae. In the gut of *L. xylina* larvae, *Enterococcus* accounted for the highest proportion of microbiota (58.05%), followed by *Acinetobacter* (6.15%) and *Weissella* (5.42%). In the gut of *L. xylina* adults, *Enterococcus* was also the most dominant microbiota (79.91%), followed by *Acinetobacter* (2.46%) and *Acidovorax* (0.83%) (Table A4).

### 3.4. The Diversity Differences for Bacterial Communities with Different Development Diets for L. xylina

**Figure 3:**
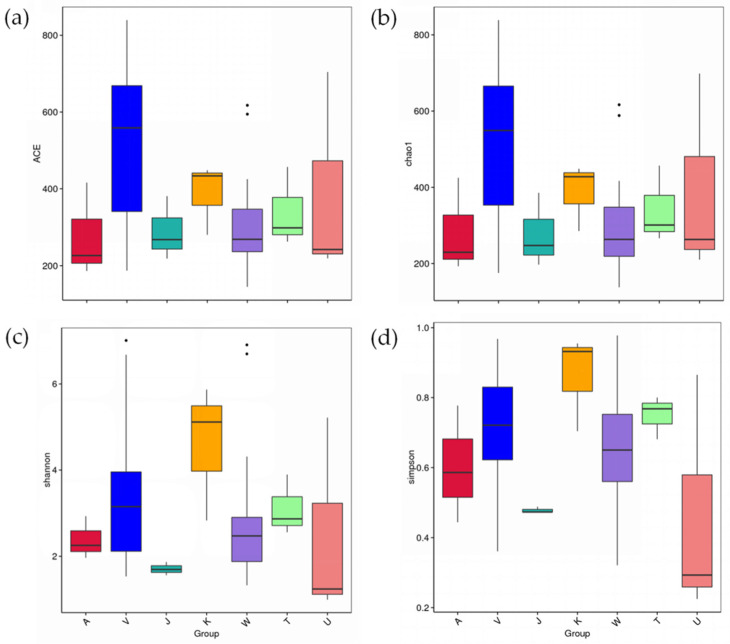
Gut bacterial diversity of *L. xylina* during development stages: (**a**) ACE index; (**b**) Chao1 index; (**c**) Shannon index; (**d**) Simpson index. Note: A, V, and J represent eggs, larvae, and adults of *L. xylina* fed on SAC, respectively; K, W, T, and U represent eggs, larvae, pupae, and adults of *L. xylina* fed on NSC, respectively. The data are presented as box-plots.

There were no significant differences between the Chao1 values of microbiota from the disinfected *L. xylina* eggs and those of microbiota from non-sterilized eggs (*t*-test, df = 4, *p* = 0.301). The gut microbiota OTU numbers for *L. xylina* fed on SAC (ACE = 521.6) during the larval stages were higher than for those fed on NSC (ACE = 308.8), while the third instar larvae of *L. xylina* fed on SAC contained the largest number of OTUs (1108 OTUs, Chao1 = 718.128). In the adult stage, there were no significant differences between the OTU numbers of *L. xylina* bacteria on NSC and those on SAC diets (*t*-test, df = 40, *p* = 0.527) (Figure 3).

**Figure 4:**
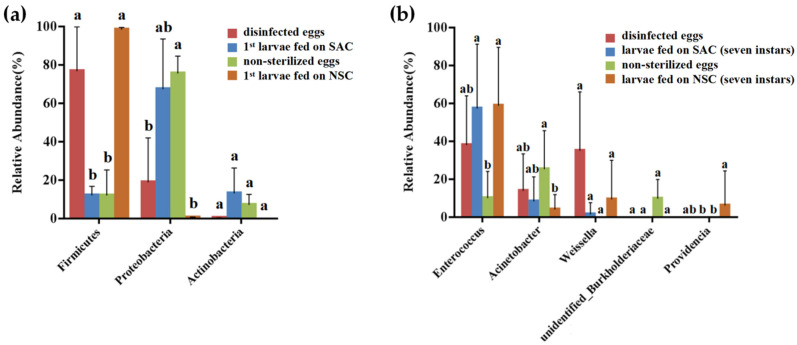
Bar chart of the phylum- and genus-level microbiota classifications for different diets (>5%): (**a**) variations in core bacterial phyla in/on the *L. xylina*’s eggs and of larvae in the first instar fed on different diets; (**b**) variations in bacterial communities in/on the *L. xylina*’s eggs and across seven instars for larvae fed on different diets at the genus level. The bars marked with different letters for the same bacterial phylum are significantly different in their abundance based on statistical analysis (*p* < 0.05).

At the phylum level, on comparing the abundance of gut microbiota of *L. xylina* fed on different diets, the most dominant microbiota in/on non-sterilized eggs was Proteobacteria (75.57%), which was significantly more abundant than Firmicutes (11.94%). The abundance of Firmicutes (98.34%) increased significantly for first instar larvae fed on NSC (*t*-test, df = 4, *p* = 0.011). In contrast, the most abundant phylum in/on disinfected eggs of *L. xylina* was Firmicutes (76.7%). The relative abundance of Firmicutes decreased significantly (*t*-test, df = 4, *p* = 0.018) for the first instar larvae fed on SAC, and Proteobacteria became the most dominant bacteria (Figure 4a). At the genus level, *Enterococcus* was the most abundant in all samples, accounting for an average of 56.7% of each sample, followed by *Acinetobacter* (7.2%) and *Weissella* (6.2%). *Enterococcus* was also the most abundant bacteria in/on disinfected eggs (37.9%), followed by *Weissella* (35%) and *Acinetobacter* (13.7%). At the phylum level, with the increase in age, the diversity of the intestinal microbiota in the NSC and SAC treatments were the same at the genus level, showing a trend of decreasing first and then increasing. There was no significant difference in the gut microbiota of *L. xylina* adults on different diets (*p* > 0.05) (Figure 4b).

**Figure 5:**
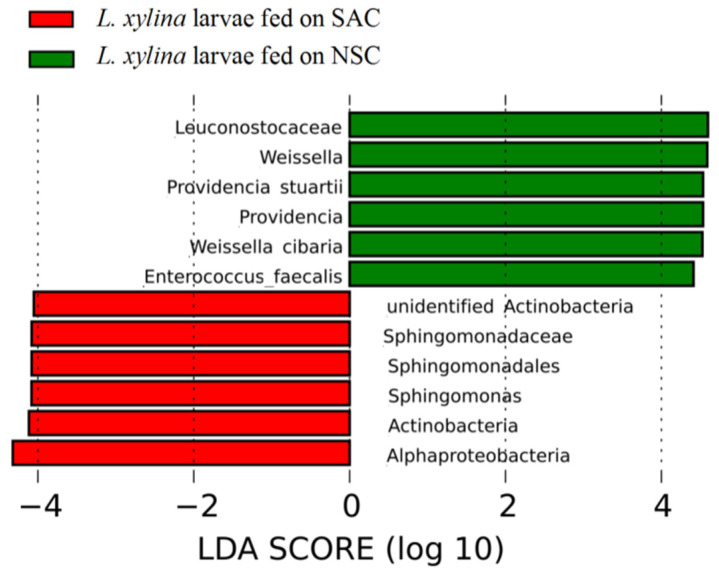
LEfSe analysis of the LDA distribution histogram of the gut microbiota across seven instars for *L. xylina* larvae fed on the SAC and NSC diets.

According to the results of the linear discriminant analysis (LDA), the main differences caused by different diet treatments are shown in Figure 5. The generic biomarkers were *Sphingomonas* in *L. xylina* larvae on the SAC diet and *Weissella* and *Providencia* in *L. xylina* larvae on the NSC diet.

## 4. Discussion

The symbiotic association between bacteria and insects has attracted extensive attention. In our study, the gut microbiota of *L. xylina* fed on different diets were significantly different, and the diversity of the gut microbiota of *L. xylina* fed on SAC (2563 OTUs) was more abundant than for *L. xylina* fed on NSC (2178 OTUs). The associated bacteria of *L. xylina* fed on SAC were extremely abundant; however, with the effects of the bacteria from the host plant and environment, the bacterial community became simpler; this might have been related to bacterial recruitment to balance the benefits and costs associated with environmental acquisition, which was associated with higher growth rates and higher metabolic costs. However, the bacteria numbers detected could be reduced because the result was the relative abundance of bacteria detected by high-throughput sequencing.

The symbiotic association was due to the influence of diet, but also may have been related to vertical transmission. As there is currently little evidence concerning the presence of insect-specific gut bacteria in/on eggs, it is difficult to determine whether bacteria in the gut is spread by vertical or horizontal transmission. The use of eggs as vectors for vertical transmission of Lepidoptera remains speculative [25]. *Acinetobacter* was the dominant microbiota for non-sterilized eggs. *Acinetobacter* was also the most dominant microbiota in/on *Cnaphalocrocis medinalis* eggs and first instar larvae [22]. However, *Enterococcus* was the dominant bacteria for disinfected eggs. Disinfecting the eggs with ethanol was not sufficient to kill all bacteria. The bacterial community structure was also affected by the varying susceptibilities of bacteria to ethanol. *Enterococcus* could live long-term on environmental surfaces, and were tolerant to heat and some alcohol preparations [26]. The relative abundance of *Acinetobacter* in the first instar larvae on the SAC diet was significantly higher than those on the NSC diet. The predominance of *Acinetobacter* may have been caused by maternal inheritance. *Acinetobacter* in the intestinal tract of fifth instar larvae of *H. armigera* shows strong esterase activity, which promotes the metabolism of the insecticide cypermethrin, thereby enhancing insect resistance [27]. Additionally, *Acinetobacter*, which is also isolated in the intestine of *Plutella xylostella* larvae, may inhibit *Phytophthora capsici* and improve the nitrogen-fixation capacity and phosphorus content [28]. The presence of diazotrophic bacteria in the gut tract of insects is helpful in promoting nitrogen absorption and enhancing immunity [29]. In the SAC treatment, larvae seemed to obtain gut microbes through vertical transmission, while in the NSC treatment, larvae seemed to obtain gut microbes through their diet and environment. The phenomenon of the vertical transmission of gut bacteria was also reported by Hosokawa and colleagues in their research on stinkbugs [4]. In *Pediculus humanus*, the intestinal commensal bacteria Candidatus *Riesia pediculicola* leave the host insect through a hole in the symbiotic spores, gather in the lateral oviducts, and enter the eggs when the eggs are discharged into the lateral oviducts [30].

In this study, in the early stages of development, the head widths of *L. xylina* larvae fed on SAC were narrower than those of larvae fed on NSC, which may have been because *L. xylina* larvae without bacteria from the host plant could not effectively obtain nutrients from the host plant. After the fifth instar, the head width growth rate for the larvae fed on the SAC diet was faster than for those fed on the NSC diet. Until the 7th instar, there were no significant differences between the respective head widths of *L. xylina* larvae fed on the two different diets. *L. xylina* had higher growth rates when fed on host plants than when fed on the artificial diet in the short term. This study suggested that *L. xylina* without bacteria derived from their diets needed more time to adjust their gut bacterial community structure and adapt to the environment [10]. The surfaces of newly deposited eggs were also a symbiotic bacteria resource for the first instar larva [31]. The destruction of the symbiotic bacteria by disinfecting the surfaces of newly deposited eggs caused the hosts to suffer from growth retardation, lower reproduction success rates, and higher mortality [32,33]. The change of the intestinal bacterial community structure from *Diabrotica virgifera virgifera* helped promote the rapid adaptation of insects in a managed ecosystem [34]. When *Pieris canidia* was treated with antibiotics for gut-associated bacteria, the host’s weight was reduced after the treatment, and the effect increased with increased concentrations of the antibiotics [35]. The gut bacterial communities resulting from NSC and SAC treatments were assessed to determine the relationships between the gut microbiota, diet, and the self-regulation ability of *L. xylina*. Changes of the gut microbiota from Lepidoptera have been determined via different factors including diet, the environment, gut physiology, and insect development stages, which could work alone or in concert [36]. There were no significant differences in the gut microbiota of *L. xylina* after the second instar, whether fed on NSC or SAC. A previous study focusing on the herbivore *Spodoptera littoralis* showed highly conservative intestinal bacterial community structures and member compositions when fed on different diets at the same developmental stages and under uniform conditions [37]. The gut microbiota of *L. xylina* shared some common taxa and similar community structures between the different diets at the same instars; this phenomenon has also been found for other herbivores [38]. The main bacteria for *L. xylina* were *Enterococcus*, *Acinetobacter*, and *Weissella* at the genus level; these bacteria have also been found present in 70% of other Lepidoptera insects [25]. *Enterococcus* comprises the largest proportion of gut microbiota of Helicoverpa armigera (Hubner), whether in the laboratory or in the field [39]. *Enterococcus* have also been found in *L. dispar* fed on different diets [40]. The gut microbiota of *L. xylina* larvae in the current study were mainly *Enterococcus*. *Enterococcus* is also the most abundant bacteria associated with two other larva types from Lepidoptera (African cotton leaf worm and *H. armigera*) [41]. *Enterococcus* is commonly found in a wide range of insect gut communities, benefiting the health and growth of the insect via various functions, including B vitamin biosynthesis, pheromone production, and the degradation of host-plant compounds [42]. Gut-associated *Enterococcus* may reduce the pH value of the gut microenvironment, enabling *L. dispar* to maintain acid-base balance [43]. *Enterococcus* may also protect the gut against pathogenic toxins that are activated in alkaline conditions by adapting the pH, which could improve gut immunity [44,45]. As a lactic acid bacterium, *Weissella* is responsible for the fermentation of food in the intestines, and directly affects the production of organic acids, esters, and alcohols.

According to the functional analysis, the functions of the gut microbiota of *L. xylina* included metabolism, genetic information processing, and environmental information processing, while secondary functions were transmembrane transport, carbohydrate metabolism, replication, and repair. The most abundant gene was related to the membrane transport function in the first instar larvae fed on NSC. The number of genes related to carbohydrate metabolism in *L. xylina* samples fed on SAC was less than for samples fed on NSC. *L. xylina* samples fed on NSC may have had greater metabolic demands. Moreover, there were more genes related to the function of endocrine and metabolic diseases and the immune system in *L. xylina* samples fed on NSC than those fed on SAC. *L. xylina* may need to adapt to more hostile environments in the wild. The increase in microorganisms in insects may increase the breadth of the insect’s feeding range [46]. Increasing the abundance of gut microbes in insects is critical, as gut commensal bacteria are key to the digestion of foods and may be particularly important for regulating the host’s pathogenicity (both positively and negatively) [47]. Gut-associated bacteria play an important role in the development of *L. xylina* larvae by facilitating the degradation of plants and other organic matter consumed by *L. xylina* larvae.

## 5. Conclusions

This study revealed the gut bacterial community structures of *L. xylina* and the relationships between gut bacteria and host insect development. The effects of diet on symbiotic microbes were also clarified in this study. The gut microbiota of *L. xylina* used vertical transmission and self-regulation to better consume the host’s nutrients.

## Figures and Tables

**Table 1:** The head capsule widths of *L. xylina* larvae fed on SAC and NAC diets.

Sample	Head Capsule Width (mm)	Coefficient Variation	Brooks Ratio	Crosby Ratio	*p* Value
A1	0.66 ± 0.175	6.6%	-	-	0.193
N1	0.80 ± 0.099	14.1%	-	-	
A2	0.99 ± 0.012	6.0%	1.500	-	0.065
N2	1.23 ± 0.066	9.4%	1.522	-	
A3	1.40 ± 0.029	5.4%	1.406	−6.3%	0.001 **
N3	1.96 ± 0.105	5.8%	1.590	4.3%	
A4	1.94 ± 0.081	9.0%	1.389	1.2%	0.000 ***
N4	3.36 ± 0.059	5.8%	1.715	7.9%	
A5	3.08 ± 0.065	6.1%	1.588	14.4%	0.000 ***
N5	4.26 ± 0.034	3.5%	1.268	−26.1%	
A6	4.90 ± 0.265	8.8%	1.591	0.1%	0.605
N6	5.17 ± 0.024	2.6%	1.213	−4.3%	
A7	6.17 ± 4.941	11.2%	1.259	−38.4%	0.005 *
N7	6.15 ± 0.091	7.1%	1.190	−1.9%	

Note: A1–A7: First to seventh instar larvae of *L. xylina* under the SAC treatment; N1–N7: first to seventh instar larvae of *L. xylina* under the NSC treatment; *p* value: the *t*-test was used to compare the differences in larvae fed on two diets at the same instar; *: *p* < 0.05; **: *p* < 0.01; ***: *p* < 0.001; data were the means ± standard error.

## Data Availability

The data presented in this study are openly available in Zenodo at https://doi.org/10.5281/zenodo.5235663.

## Appendix A

**Table A1:** The body lengths of *L. xylina* larvae fed on SAC and NAC.

Sample	Body Length (mm)	Coefficient Variation	Brooks’ Ratio	Crosby’s Ratio	*p* Value
A1	3.61 ± 0.195	14.2%	-	-	0.048 *
N1	5.61 ± 0.768	13.7%	-	-	
A2	5.38 ± 0.187	11.1%	1.489	-	0.006 **
N2	7.67 ± 3.333	7.5%	1.366	-	
A3	7.64 ± 0.188	12.1%	1.420	−4.6%	0.046 *
N3	10.00 ± 0.837	18.7%	1.304	−4.5%	
A4	11.11 ± 0.462	9.4%	1.455	2.5%	0.000 ***
N4	14.71 ± 0.610	19.0%	1.471	9.9%	
A5	19.96 ± 0.334	10.1%	1.796	23.4%	0.324
N5	18.84 ± 1.055	24.4%	1.281	−13.0%	
A6	31.38 ± 0.507	12.5%	1.572	−12.5%	0.000 ***
N6	21.97 + 0.613	15.5%	1.166	−9.0%	
A7	43.43 ± 0.717	15.5%	1.384	−12.0%	0.000 ***
N7	29.74 ± 1.395	22.5%	1.354	1.6%	

Note: A1–A7: first to seventh instars of larvae of *L. xylina* under the SAC treatment; N1–N7: first to seventh instars of larvae of *L. xylina* under the NSC treatment; *p* values: the *t*-test was used to compare the differences in larvae fed on two diets at the same instar; *: *p* < 0.05; **: *p* < 0.01; ***: *p* < 0.001; data are the means ± standard error.

**Table A2:** The α diversity index values of the gut microbiota of *L. xylina* samples fed on SAC and NSC, calculated at the same level, 97%.

Development	OTUs	Chao1	Shannon-Wiener Index	Simpson Index	Goods Coverage
Egg	974	334.744	3.494	0.733	0.999
1st instar larvae	1245	418.548	3.062	0.664	0.998
2nd instar larvae	1140	402.394	3.189	0.706	0.998
3rd instar larvae	1258	483.053	2.807	0.664	0.996
4th instar larvae	1389	531.653	4.484	0.815	0.996
5th instar larvae	1250	425.975	3.392	0.665	0.998
6th instar larvae	619	279.847	2.101	0.586	0.998
7th instar larvae	952	318.271	2.586	0.666	0.998
Adult	964	333.503	2.096	0.470	0.998

**Table A3:** The top 10 phyla with the highest relative abundance levels in the gut microbiota of *L. xylina* during different developmental stages.

Phylum Level	Egg	1st Instar Larvae	2nd Instar Larvae	3rd Instar Larvae	4th Instar Larvae	5th Instar Larvae	6th Instar Larvae	7th Instar Larvae	Pupa	Adult
Firmicutes	44.33	55.20	80.01	68.03	42.97	74.88	90.29	79.30	63.94	86.09
Proteobacteria	47.23	33.95	13.40	27.64	39.09	14.04	7.29	11.43	24.95	9.92
Actinobacteria	3.73	6.58	2.51	1.25	2.63	1.06	0.10	0.50	0.46	1.17
Cyanobacteria	0.27	0.20	0.35	0.21	4.67	1.76	1.44	4.82	0.75	0.28
Bacteroidetes	2.74	1.12	2.14	1.82	6.18	5.45	0.18	2.97	8.78	2.09
Verrucomicrobia	0.05	0.03	0.07	0.05	1.13	0.09	0.00	0.01	0.01	0.04
Chloroflexi	0.02	0.97	0.06	0.01	0.12	0.13	0.00	0.04	0.03	0.01
Acidobacteria	0.10	0.71	0.12	0.04	0.34	0.36	0.01	0.04	0.02	0.02
Gemmatimonadetes	0.02	0.36	0.05	0.01	0.02	0.08	0.00	0.01	0.01	0.02
Euryarchaeota	0.00	0.00	0.02	0.00	0.36	0.07	0.00	0.01	0.00	0.00
Others	1.51	0.88	1.27	0.92	2.50	2.10	0.68	0.88	1.04	0.36

**Table A4:** The top 10 genera with the highest relative abundance levels for the gut microbiota of *L. xylina* during different developmental stages.

Phylum Level	Egg	1st Instar Larvae	2nd Instar Larvae	3rd Instar Larvae	4th Instar Larvae	5th Instar Larvae	6th Instar Larvae	7th Instar Larvae	Pupa	Adult
*Enterococcus*	23.98	34.35	57.67	56.49	28.89	63.62	89.58	75.75	61.02	79.91
*Providencia*	0.00	0.01	0.01	21.31	0.02	0.02	0.01	0.01	0.00	0.01
*Weissella*	17.48	14.94	16.30	1.40	0.05	5.08	0.11	0.09	0.00	0.05
*Acinetobacter*	19.61	16.77	3.94	1.22	2.14	5.42	5.33	8.24	20.64	2.46
unidentified *Cyanobacteria*	0.25	0.20	0.34	0.20	4.66	1.76	1.44	4.81	0.74	0.28
unidentified Burkholderiaceae	4.87	0.00	0.00	0.06	0.12	0.00	0.00	0.00	0.00	0.00
*Staphylococcus*	0.03	1.45	2.57	6.90	0.16	0.12	0.03	0.02	0.06	0.24
*Empedobacter*	1.50	0.03	0.01	0.02	0.01	0.00	0.00	2.23	6.97	0.01
*Acidovorax*	1.10	6.04	0.79	0.28	0.82	0.34	0.35	0.46	0.69	0.83
*Sphingomonas*	1.10	6.30	0.35	0.86	2.18	1.05	0.48	0.31	0.15	0.24
Others	30.07	19.91	18.01	11.27	60.95	22.60	2.66	8.07	9.72	15.97

## References

[1] Liu Y., Shen Z., Yu J., Li Z., Liu X., Xu H. (2020). Comparison of gut bacterial communities and their associations with host diets in four fruit borers. Pest Manag. Sci..

[2] Zhang J.H., Yu N., Xu X.X., Liu Z.W. (2018). Community structure, dispersal ability and functional profiling of microbiome existing in fat body and ovary of the brown planthopper, *Nilaparvata lugens*. Insect Sci..

[3] Philipp E., Moran N.A. (2013). The gut microbiota of insects—Diversity in structure and function. FEMS Microbiol. Rev..

[4] Hosokawa T., Kikuchi Y., Meng X.Y., Fukatsu T. (2005). The making of symbiont capsule in the plataspid stinkbug *Megacopta punctatissima*. FEMS Microbiol. Ecol..

[5] Cheng D., Guo Z., Riegler M., Xi Z., Liang G., Xu Y. (2017). Gut symbiont enhances insecticide resistance in a significant pest, the oriental fruit fly *Bactrocera dorsalis* (Hendel). Microbiome.

[6] Chen B.S., Du K.Q., Sun C., Arunprasanna V., Liang X., Li Y., Wang B.H., Lu X.M., Li L.J., Shao Y.Q. (2018). Gut bacterial and fungal communities of the domesticated silkworm (*Bombyx mori*) and wild mulberry-feeding relatives. ISME J..

[7] Akiko S., Dubreuil G., David G., Jean C.S. (2014). Plant–insect interactions under bacterial influence: Ecological implications and underlying mechanisms. J. Exp. Bot..

[8] Su L.J., Yang L.L., Huang S., Li Y., Su X.Q., Wang F.Q., Bo C.P., Wang E.T., Song A.D. (2016). Variation in the gut microbiota of termites (*Tsaitermes ampliceps*) against different diets. Appl. Biochem. Biotechnol..

[9] Zeng J.Y., Shi J.H., Guo J.X., Shi Z.B., Zhang G.C., Zhang J. (2020). Variation in the pH of experimental diets affects the performance of *Lymantria dispar asiatica* larvae and its gut microbiota. Arch. Insect Biochem..

[10] Shen T.C., Tseng C.M., Guan L.C., Hwang S.Y. (2006). Performance of *Lymantria xylina* (Lepidoptera: Lymantriidae) on artificial and host plant diets. J. Econ. Entomol..

[11] Sree K.S., Varma A. (2015). Biocontrol of Lepidopteran Pests.

[12] Chao J.T., Paul W.S., Fan Y.B., Lu S.S. (1996). Host plants and infestation of casuarina moth *Lymantria xylina* in Taiwan. Taiwan For. Sci..

[13] Wang R., Zhang Z.H., Hu X., Wu S.Q., Wang J.D., Zhang F.P. (2018). Molecular detection and genetic diversity of casuarina moth, *Lymantria xylina* (Lepidoptera: Erebidae). J. Insect Sci..

[14] Mitter C., Davis D.R., Cummings M.P. (2017). Phylogeny and evolution of Lepidoptera. Annu. Rev. Entomol..

[15] Hu X., Wang C., Chen H., Ma J. (2013). Differences in the structure of the gut bacteria communities in development stages of the Chinese white pine beetle (*Dendroctonus armandi*). Int. J. Mol. Sci..

[16] Yang M.H., Zhang J.T., Zong S.X., Luo Y.Q., Niu H.L., Zhang B. (2012). Determination of the larval instar number of the carpenter moth *Holcocerus vicarius* (Lepidoptera: Cossidae). Acta Entomol. Sin..

[17] Loerch C.R., Cameron E.A. (1983). Determination of larval instars of the bronze birch borer, *Agrilus anxius* (Coleoptera: Buprestidae). Ann. Entomol. Soc. Am..

[18] Ablhauer K.P., Wemheuer B., Daniel R., Meinicke P. (2015). Tax4Fun: Predicting functional profiles from metagenomic 16S rRNA data. Bioinformatics.

[19] Martin M. (2011). Cutadapt removes adapter sequences from high-throughput sequencing reads. EMBnet J..

[20] Torbjørn R., Tomáš F., Nichols B., Quince C., Frédéric M. (2016). VSEARCH: A versatile open source tool for metagenomics. PeerJ.

[21] Brian J.H., Dirk G., Ashlee M.E., Mike F., Doyle V.W., Georgia G., Dawn C., Diana T., Sarah K.H., Erica S. (2011). Chimeric 16S rRNA sequence formation and detection in Sanger and 454-pyrosequenced PCR amplicons. Genome Res..

[22] Wang Q., Garrity G.M., Tiedje J.M., Cole J.R. (2007). Naive Bayesian classifier for rapid assignment of rRNA sequences into the new bacterial taxonomy. Appl. Environ. Microb..

[23] Yang Y.G., Liu X.G., Xu H.X., Liu Y.H., Panna A., Muhammad A.B., Lu Z.X. (2020). The abundance and diversity of gut bacteria of rice leaffolder *Cnaphalocrocis medinalis* (Guenée) across life stages. J. Asia-Pac. Entomol..

[24] Edgar R.C. (2013). UPARSE: Highly accurate OTU sequences from microbial amplicon reads. Nat. Methods.

[25] Luis R.P.V., Enric F., Martin K., Monika H., Nina E.F. (2018). Bacterial symbionts in Lepidoptera: Their diversity, transmission, and impact on the host. Front. Microbiol..

[26] Arias C.A., Murray B.E. (2012). The rise of the *Enterococcus*: Beyond vancomycin resistance. Nat. Rev. Microbiol..

[27] Malhotra J., Dua A., Saxena A., Sangwan N., Lal R. (2012). Genome sequence of *Acinetobacter* sp. strain HA, isolated from the gut of the polyphagous insect pest *Helicoverpa Armigera*. J. Bacteriol..

[28] Indiragandhi P., Anandham R., Madhaiyan M., Sa T.M. (2008). Characterization of plant growth–promoting traits of bacteria isolated from larval guts of diamondback moth *Plutella xylostella* (Lepidoptera: Plutellidae). Curr. Microbiol..

[29] Dillon R.J., Dillon V.M. (2004). The gut bacteria of insects: Nonpathogenic interactions. Annu. Rev. Entomol..

[30] Eberle M.W., McLean D.L. (1983). Observation of symbiote migration in human body lice with scanning and transmission electron microscopy. Can. J. Microbiol..

[31] Salem H., Florez L., Gerardo N., Kaltenpoth M. (2015). An out-of-body experience: The extracellular dimension for the transmission of mutualistic bacteria in insects. Proc. R. Soc. B.

[32] Kikuchi Y., Hosokawa T., Nikoh N., Meng X.Y., Kamagata Y., Fukatsu T. (2009). Host-symbiont co-speciation and reductive genome evolution in gut symbiotic bacteria of acanthosomatid stinkbugs. BMC Biol..

[33] Salem H., Kreutzer E., Sudakaran S., Kaltenpoth M. (2013). Actinobacteria as essential symbionts in firebugs and cotton stainers (Hemiptera, Pyrrhocoridae). Environ. Microbiol..

[34] Chu C.C., Joseph L.S., Matías J.C., Jorge A.Z., Manfredo J.S. (2013). Gut bacteria facilitate adaptation to crop rotation in the western corn rootworm. Proc. Natl. Acad. Sci. USA.

[35] Wang Y., Zhu J.Q., Fang J., Shen L., Ma S.J., Zhao Z.M., Yu W.D., Jiang W.B. (2020). Diversity, composition and functional inference of gut microbiota in indian cabbage white *Pieris canidia* (Lepidoptera: Pieridae). Life.

[36] Staudacher H., Kaltenpoth M., Breeuwer J.A., Menken S.B., Heckel D.G., Groot A.T. (2016). Variability of bacterial communities in the moth *Heliothis virescens* indicates transient association with the host. PLoS ONE.

[37] Awmack C.S., Leather S.R. (2002). Host plant quality and fecundity in herbivorous insects. Annu. Rev. Entomol..

[38] Xia X., Gurr G.M., Vasseur L., Zheng D., Zhong H., Qin B., Lin J.H., Wang Y., Song F.Q., Li Y. (2017). Metagenomic sequencing of diamondback moth gut microbiome unveils key holobiont adaptations for herbivory. Front. Microbiol..

[39] Xiang H., Wei G.F., Jia S.H., Huang J.H., Miao X.X., Zhou Z.H., Zhao L.P., Huang Y.P. (2007). Microbial communities in the larval midgut of laboratory and field populations of cotton bollworm (*Helicoverpa armigera*). Can. J. Microbiol..

[40] Broderick N.A., Raffa K.F., Goodman R.M., Handelsman J. (2004). Census of the bacterial community of the gypsy moth larval midgut by using culturing and culture-independent methods. Appl. Environ. Microbiol..

[41] Tang X.H., Dalial F., Heiko V., Ping L.Y., Shao Y.Q., Erika A.C., Gary A., Martin W., David G.H., Wilhelm B. (2012). Complexity and variability of gut commensal microbiota in polyphagous Lepidopteran larvae. PLoS ONE.

[42] Breznak J.A. (1982). Intestinal microbiota of termites and other xylophagous insects. Annu. Rev. Microbiol..

[43] Dow J.A. (1992). pH gradients in lepidopteran midgut. J. Exp. Biol..

[44] Li P.H., Niu Q., Wei Q.T., Zhang Y.Q., Ma X., Sung W.K., Ling M.X., Huang R.H. (2017). Microbial shifts in the porcine distal gut in response to diets supplemented with *Enterococcus Faecalis* as alternatives to antibiotics. Sci. Rep..

[45] Shao Y., Chen B., Sun C., Ishida K., Hertweck C., Boland W. (2017). Symbiont-derived antimicrobials contribute to the control of the lepidopteran gut microbiota. Cell Chem. Biol..

[46] Lundgren J.G., Lehman R. (2010). Bacterial gut symbionts contribute to seed digestion in an omnivorous beetle. PLoS ONE.

[47] Schmid R.B., Michael L.R., Brözel V.S., Lundgren J.G. (2015). Gut bacterial symbiont diversity within beneficial insects linked to reductions in local biodiversity. Ann. Entomol. Soc. Am..

